# START: a system for flexible analysis of hundreds of genomic signal tracks in few lines of SQL-like queries

**DOI:** 10.1186/s12864-017-4071-1

**Published:** 2017-09-22

**Authors:** Xinjie Zhu, Qiang Zhang, Eric Dun Ho, Ken Hung-On Yu, Chris Liu, Tim H. Huang, Alfred Sze-Lok Cheng, Ben Kao, Eric Lo, Kevin Y. Yip

**Affiliations:** 1 Department of Computer Science, The University of Hong Kong, Pokfulam Road, Hong Kong, Hong Kong; 2 School of Computing, Hong Kong Polytechnic University, Hung Hom, Kowloon Hong Kong; 3 Department of Computer Science and Engineering, The Chinese University of Hong Kong, Shatin, New Territories Hong Kong; 4 Department of Anatomical and Cellular Pathology, The Chinese University of Hong Kong, Shatin, New Territories Hong Kong; 5 Department of Molecular Medicine, University of Texas Health Science Center at San Antonio, San Antonio, Texas USA; 6 School of Biomedical Sciences, The Chinese University of Hong Kong, Shatin, New Territories Hong Kong; 7 Hong Kong Bioinformatics Centre, The Chinese University of Hong Kong, Shatin, New Territories Hong Kong; 8 CUHK-BGI Innovation Institute of Trans-omics, The Chinese University of Hong Kong, Shatin, New Territories Hong Kong; 9 Hong Kong Institute of Diabetes and Obesity, The Chinese University of Hong Kong, Shatin, New Territories Hong Kong

**Keywords:** Human genomics, Signal tracks, Data analysis

## Abstract

**Background:**

A genomic signal track is a set of genomic intervals associated with values of various types, such as measurements from high-throughput experiments. Analysis of signal tracks requires complex computational methods, which often make the analysts focus too much on the detailed computational steps rather than on their biological questions.

**Results:**

Here we propose Signal Track Query Language (STQL) for simple analysis of signal tracks. It is a Structured Query Language (SQL)-like declarative language, which means one only specifies *what* computations need to be done but not *how* these computations are to be carried out. STQL provides a rich set of constructs for manipulating genomic intervals and their values. To run STQL queries, we have developed the Signal Track Analytical Research Tool (START, http://yiplab.cse.cuhk.edu.hk/start/), a system that includes a Web-based user interface and a back-end execution system. The user interface helps users select data from our database of around 10,000 commonly-used public signal tracks, manage their own tracks, and construct, store and share STQL queries. The back-end system automatically translates STQL queries into optimized low-level programs and runs them on a computer cluster in parallel. We use STQL to perform 14 representative analytical tasks. By repeating these analyses using bedtools, Galaxy and custom Python scripts, we show that the STQL solution is usually the simplest, and the parallel execution achieves significant speed-up with large data files. Finally, we describe how a biologist with minimal formal training in computer programming self-learned STQL to analyze DNA methylation data we produced from 60 pairs of hepatocellular carcinoma (HCC) samples.

**Conclusions:**

Overall, STQL and START provide a generic way for analyzing a large number of genomic signal tracks in parallel easily.

**Electronic supplementary material:**

The online version of this article (doi:10.1186/s12864-017-4071-1) contains supplementary material, which is available to authorized users.

## Background

The rapid development of new applications of high-throughput sequencing and the sharp reduction of cost have made it common to produce large amounts of sequencing data that measure a variety of biological signals in a single study. For instance, large-scale disease studies can involve the sequencing of hundreds or even thousands of disease and control samples [1]. Major collaborative projects such as Encyclopedia of DNA Elements (ENCODE) [2] and Roadmap Epigenomics [3] have performed tens of thousands of high-throughput sequencing experiments that survey the genomes, transcriptomes and epigenomes of a large number of samples, creating rich and complex sets of data.

After standard data processing, sequencing data are commonly represented as signal tracks. A signal track is a set of genomic intervals each associated with a signal value. Depending on the analytical needs, the intervals can be defined in various ways. For example, when the data from a ChIP-seq (chromatin immunoprecipitation followed by high-throughput sequencing) experiment are represented as a signal track, at the basic level, each interval corresponds to a single genomic location and the associated value is the number of aligned reads that cover the location. At the next level, one could use the distribution of signal values to define signal peaks, and consider each peak as an interval with a fixed value of one (which means “present”) or a value that indicates the enrichment score of the peak as compared to control. One could also use a gene annotation set to define intervals of interest (e.g., promoters), and compute the average number of covering reads at each interval as its signal value. In each of these three cases, the ChIP-seq data are represented by a signal track. The generality of representing high-throughput sequencing data by signal tracks is exemplified by its prevalent use in genome browsers for displaying many types of sequencing data.

Analysis of signal tracks usually involves multiple steps. Typical operations at each step include selection of intervals based on certain criteria, comparison of intervals from the same or different tracks, and aggregation of multiple intervals to form new intervals. There are software tools for particular types of operation, and pipelines can be set up by writing scripts that invoke the different tools and convert the outputs of one tool into the inputs of another [4–6].

As the volume and complexity of signal track data have both increased dramatically in recent years, this paradigm of data analysis is facing several challenges. First, many existing tools have a fixed set of functions. When they do not exactly match the needs of an analytical pipeline, one would need to modify a tool or implement a new one. Second, pipelines are usually developed in an imperative language. Researchers are required to specify the detailed computational steps, which could distract him/her from focusing on the biological questions. Third, in order to perform analysis efficiently, a researcher needs to decide on proper data structures, algorithms and parallel execution environments, which impose a strong requirement on his/her computational backgrounds.

With a goal of providing a single platform that can support a large variety of analytical needs, here we describe the Signal Track Query Language (STQL) that we specifically designed for signal track data analysis. It is a declarative language with a syntax similar to the Structured Query Language (SQL) commonly used in relational database systems, which makes STQL easy to learn. Users only need to specify what operations they want to perform using some high-level constructs, but not the detailed steps of how these operations are to be performed, thereby allowing them to focus on the analytical goals rather than the technical details.

To demonstrate the broad applications of STQL, we have implemented a system for executing STQL queries called Signal Track Analytical Research Tool (START, http://yiplab.cse.cuhk.edu.hk/start/). It contains a Web interface that guides users to construct STQL queries, and provides example queries for various types of data analysis. At the back end, the submitted queries are automatically translated into executable programs, which are then run on a cluster of machines in parallel. START provides a variety of pre-loaded public data that facilitate integrated analysis of both public and private data, including data from ChromHMM [7], dbSNP [8], ENCODE, FANTOM5 (Functional Annotation of The Mammalian Genome Phase 5) [9], RoadMap Epigenomics, UCSC Genome Browser [10] and Yip et al. [11]. START also provides storage for both users’ data files and executed queries, allows sharing of queries among users, and contains features for protecting security and data privacy. Users who want to execute STQL queries locally on their own machines can download our installable package, with a detailed installation guide at https://github.com/stql/start/wiki/Install-START-in-your-own-cluster describing the steps for installing the package, pre-processing data, loading signal tracks, organizing the tracks and using the system.

The flexibility of analyzing genomic data with a query language has been clearly demonstrated in a number of recent studies [5, 12–16]. Most of these languages were designed for raw sequencing reads and cannot be used for analyzing signal tracks. GenoMetric Query Language [5] is a language designed for signal track analysis. Compared to this language, STQL provides a large set of interval comparison relations that help simplify queries, constructs for manipulating signal values (based on the **EACH MODEL** and **TOTAL MODEL**), several types of loop statements, complex queries such as those involving sub-queries, the **discretize** operation for creating non-overlapping intervals, and has an SQL-like design that makes it easier to learn for people with SQL experience.

In the followings we describe the different components of STQL and how it can be used to analyze genomic signal tracks. We present illustrative example queries that correspond to commonly performed analytical operations. These example queries include both simple ones that show individual language features of STQL, as well as composite ones that involve multiple steps.

To evaluate the correctness, simplicity and execution efficiency of STQL, we used several other popular approaches to carry out the same analytical tasks, including bedtools [6], Galaxy [4] and custom Python scripts we specifically wrote for these tasks. We show that many of these tasks are most easily carried out by using STQL, and for tasks involving large data files, the transparent parallelization of STQL provided by START leads to significant speed-ups.

We further demonstrate the usability of STQL by describing how a biologist with minimal training in computer programming self-learned STQL to identify genes affected by differential promoter methylation by integrating private sequencing data from 60 pairs of hepatocellular carcinoma (HCC) case-control samples and public signal tracks. The STQL queries written serve as a succinct log of the analyses taken, allowing anyone to reproduce the same results and apply the pipeline to other data sets easily.

## Implementation

### Data model of STQL

In STQL, each track is composed of a set of intervals all with the same attributes (possibly with null values). Each interval contains four mandatory attributes, namely its chromosome (‘.chr’), starting position (‘.chrstart’, one-based inclusive), ending position (‘.chrend’, inclusive), and value (‘.value’). Each signal track can define any number of additional attributes for its intervals. For example, a.strand attribute can be defined to contain the strand of each interval, with values ‘ + ’, ‘ −’ and ‘.’ for the positive strand, negative stand, and don’t care/not available, respectively. Each interval is therefore equivalent to a tuple in a relational table with a list of single-valued attributes.

### Basic constructs in STQL

The formal grammar of STQL is given in the STQL grammar rules section. Basically, each STQL query contains three main parts, namely a SELECT clause for specifying interval attributes to be included in the results, a FROM clause for the signal tracks to query from, and an optional WHERE clause for defining criteria for filtering intervals. For example, the following query returns all attributes of the intervals on chromosome 1 from a signal track T:



If the query result includes all the four mandatory attributes, it will be considered a signal track itself and can be used as an input track of another query.

#### The SELECT clause

The SELECT clause includes a comma-separated list of attributes of the queried intervals to be returned, which can include both the four mandatory attributes and any of the additional attributes defined for the tracks involved. STQL also supports other syntactic constructs commonly used in the SELECT clause of SQL, such as the DISTINCT keyword for removing duplicates, standard arithmetic operations (addition, subtraction, multiplication and division), and the AS keyword for renaming attributes. As in SQL, if the signal track from which an attribute comes is unambiguous, the attribute can be listed without stating the track name. For example, the following query returns the set of distinct interval lengths for the intervals in a track T:



Since interval lengths are commonly queried in analysis tasks, STQL also defines a short-hand (“syntactic sugar”) for it, allowing the above query to be written in a simpler form:



#### The FROM clause

The FROM clause contains a comma-separated list of signal tracks to query from. Each listed track can be an existing signal track in the database, a nested query (described below), or a track dynamically generated using one of the track operations to be described in the section on advanced constructs.

In STQL, conceptually a Cartesian product of the listed tracks is performed in a chromosome-by-chromosome manner, since intervals from different chromosomes are seldom directly compared. For example, suppose we have the following two tracks T_1_ and T_2_:

**Table:** 

T_1_			
chr	chrstart	chrend	value
chr1	101	200	10
chr1	201	300	20
chr2	301	400	30

T_2_			
chr	chrstart	chrend	value
chr1	401	500	40
chr2	501	600	50
chr3	601	700	60

Suppose the following query is issued to identify all pairs of intervals on the same chromosome from the two tracks:



The query results will be as follows:

**Table:** 

chr	chrstart	chrend	value	chr2	chrstart2	chrend2	value2
chr1	101	200	10	chr1	401	500	40
chr1	201	300	20	chr1	401	500	40
chr2	301	400	30	chr2	501	600	50

The results do not involve any intervals from chromosome 3, because T_1_ does not contain any interval on this chromosome. One could also use LEFT JOIN, RIGHT JOIN and OUTER JOIN to include intervals on chromosomes that appear only in the first, second or either of the two joining tracks. For example, suppose RIGHT JOIN is used in the previous query:



Then the query results will be as follows:

**Table:** 

chr	chrstart	chrend	value	chr2	chrstart2	chrend2	value2
chr1	101	200	10	chr1	401	500	40
chr1	201	300	20	chr1	401	500	40
chr2	301	400	30	chr2	501	600	50
NULL	NULL	NULL	NULL	chr3	601	700	60

In our actual implementation, instead of performing the costly Cartesian product followed by filtering the pairs that satisfy the conditions specified in the WHERE clause, the intervals in each track are sorted and compared directly to produce the list of pairs that satisfy the conditions.

As in SQL, if a signal track T appears in the FROM clause, writing T.chr means the chromosome of an instance (i.e., an interval) on track T. To make the meaning of the query clearer, one could give an alias to each track by appending the alias after the track name in the FROM clause. For instance, the interval length example given above can also be written as follows:



By using the alias *T* *I* *n* *t*, it is clear that the query returns the lengths of the intervals in the signal track as its results. We recommend adding aliases in this way since the resulting queries are easier to understand, but syntactically the aliases are not mandatory.

#### The WHERE clause

The WHERE clause contains a logical expression that specifies which intervals should be kept in the results. The logical expression can be composed of primitive expressions joined together by standard logical operators AND, OR and NOT. As in SQL, each primitive expression can involve a mathematical equality or inequality (e.g., **length**(TInt) < 1000). In addition, since in many analysis tasks, different genomic intervals are compared to determine the ones to be included in the final results, a list of common relations are defined in STQL to express the positional relationships among intervals. Table 1 lists the formal definitions of these interval relations, and provides an example use of each relation. If additional relations are needed in a certain task, they can be constructed in STQL queries using the primitive constructs.

**Table 1:** Relations defined in STQL for comparing different intervals

Relation	Definition	Example use
I_1_ **coincides** **with** I_2_	I_1_.chr = I_2_.chr and I_1_.chrstart = I_2_.chrstart and I_1_.chrend = I_2_.chrend	From the tracks of two replicated experiments where each interval stores the average signal of a genomic bin, find out the bins with values in both experiments
I_1_ **overlaps** **with** I_2_	I_1_.chr = I_2_.chr and I_1_.chrstart ≤ I_2_.chrend and I_1_.chrend ≥ I_2_.chrstart	From a track of intervals that represent a type of signal, find out those that overlap the promoters defined as intervals in another track
I_1_ **contains** I_2_	I_1_.chr = I_2_.chr and I_1_.chrstart ≤ I_2_.chrstart and I_1_.chrend ≥ I_2_.chrend	From a track of intervals that represent transcription factor binding sites, find out those that contain single nucleotide variants defined as intervals in another track
I_1_ **is within** I_2_	I_1_.chr = I_2_.chr and I_1_.chrstart ≥ I_2_.chrstart and I_1_.chrend ≤ I_2_.chrend	From a track of intervals that represent genes, find out those that are contained by haplotype blocks defined as intervals in another track
I_1_ **is adjacent** **to** I_2_	I_1_.chr = I_2_.chr and (I_1_.chrend + 1 = I_2_.chrstart or I_1_.chrstart − 1 = I2.chrend)	From a track of intervals that represent different sequence elements, find out the flanking exons of an intron
I_1_ **is prefix of** I_2_	I_1_.chr = I_2_.chr and I_1_.chrstart = I_2_.chrstart and I_1_.chrend ≤ I_2_.chrend	From a track of intervals that represent genes and their sub-elements, find out the first exon of each gene on the positive strand
I_1_ **is suffix of** I_2_	I_1_.chr = I_2_.chr and I_1_.chrstart ≥ I_2_.chrstart and I_1_.chrend = I_2_.chrend	From a track of intervals that represent genes and their sub-elements, find out the first exon of each gene on the negative strand
I_1_ **precedes** I_2_	I_1_.chr = I_2_.chr and I_1_.chrend < I_2_.chrstart	Ordering any type of intervals on the same chromosome
I_1_ **follows** I_2_	I_1_.chr = I_2_.chr and I_1_.chrstart > I_2_.chrend	Ordering any type of intervals on the same chromosome
I_1_ **is upstream** **of** I_2_	I_1_.chr = I_2_.chr and ((I_2_.strand = ‘ + ’ and I_1_.strand = ‘ + ’ and I_1_ **precedes** I_2_) or (I_2_.strand = ‘ + ’ and I_1_.strand = ‘.’ and I_1_ **precedes** I_2_) or (I_2_.strand = ‘ −’ and I_1_.strand = ‘ −’ and I_1_ **follows** I_2_) or (I_2_.strand = ‘ −’ and I_1_.strand = ‘.’ and I_1_ **follows** I_2_))	From a track of intervals that represent transcripts, define their promoter regions
I_1_ **is downstream of** I_2_	I_1_.chr = I_2_.chr and ((I_2_.strand = ‘ + ’ and I_1_.strand = ‘ + ’ and I_1_ **follows** I_2_) or (I_2_.strand = ‘ + ’ and I_1_.strand = ‘.’ and I_1_ **follows** I_2_) or (I_2_.strand = ‘ −’ and I_1_.strand = ‘ −’ and I_1_ **precedes** I_2_) or (I_2_.strand = ‘ −’ and I_1_.strand = ‘.’ and I_1_ **precedes** I_2_))	From a track of intervals that represent sequence motifs, find out their downstream sequence elements defined as intervals in another track

The input intervals of these relations can be intervals selected from a signal track or constant intervals specified in the format “[<*chr*>, <*chrstart*>, <*chrend*>]” such as “[chr1, 100, 200]”.

Among these interval relations, **is upstream of** and **is downstream of** have the most complex definitions since they involve strand information. As in the usual sense, one can define an interval I_1_ to be upstream/downstream of another interval I_2_ only if the strand of I_2_ is known and the strand of I_1_ is either the same as I_2_ or is not available.

Since it is common to analyze genomic distances, there is also a function **distance**() defined in STQL for computing the distance between two genomic intervals in the WHERE clause:

$${{} \begin{aligned} \mathbf{distance}(\mathrm{I}_{1}, \mathrm{I}_{2}) = \left\{ \begin{array}{ll} \mathrm{I}_{2}.\text{chrstart} - \mathrm{I}_{1}.\text{chrend} & \text{if}\ \mathrm{I}_{1}\ \mathbf{precedes}\ \mathrm{I}_{2} \\ 0 & \text{if}\ \mathrm{I}_{1}\ \mathbf{overlaps~with}\ \mathrm{I}_{2}\\ \mathrm{I}_{1}.\text{chrstart} - \mathrm{I}_{2}.\text{chrend} & \text{if}\ \mathrm{I}_{1}\ \mathbf{follows}\ \mathrm{I}_{2}\\ \text{NaN} &\text{if } \text{I}_{\text{1}}.\text{chr} \neq \text{I}_{\text{2}}.\text{chr} \end{array} \right. \end{aligned}} $$

One frequently used operation more difficult to define using the primitive constructs is finding out the interval(s) closest to a given interval. In STQL, the **is closest to each** relation is defined for this purpose, as shown in the following example:



In this example, for each interval in T_2_, we find its closest interval among all intervals in T_1_. The result can contain zero intervals (if no intervals in T_1_ are on that chromosome), one interval, or more than one interval (if multiple intervals in T_1_ are of exactly the same closest distance from it).

#### Other optional clauses

Similar to SQL, STQL provides a GROUP BY clause for grouping intervals and performing aggregations (**COUNT()**, **SUM()**, **AVG()**, **MIN()** and **MAX()**) for each group, and an ORDER BY clause for ordering the selected intervals. For example, the following query counts the number of intervals with a value larger than 10 on each chromosome, with the resulting counts sorted in ascending order:



The basic constructs described above are sufficient for many simple analyses. On the other hand, some analyses can be more easily performed with the help of additional constructs. We next describe these advanced constructs defined in STQL.

### Advanced constructs in STQL

#### Creating a new track from an existing track

**Fig. 1:**
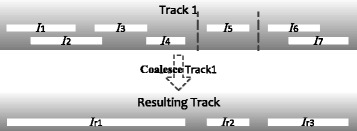
An example that illustrates the coalesce operator. I_1_.. I_7_ are intervals in the input track that are allowed to overlap with or be adjacent to each other, while I_r1_..I_r3_ are the non-overlapping, non-adjacent intervals in the resulting track after the coalesce operation. I_r1_ is formed by merging I_1_, I_2_, I_3_ and I_4_, which occupy a contiguous block of genomic locations. I_r2_ is formed by I_5_ alone, which does not overlap with or is adjacent to any other input intervals. I_r3_ is formed by merging I_6_ and I_7_

In an analysis pipeline, it is common for an intermediate step to create small intervals that can overlap or be adjacent to each other. These small regions are subsequently merged into longer regions in later steps. For example, suppose in an analysis step individual transcriptional enhancers are identified, and in the next step the overlapping or adjacent enhancers are to be merged to form potential super enhancers [17]. This type of interval merging can be performed by using the **coalesce** construct, which groups each set of transitively overlapping/adjacent intervals into a single interval, where the starting and ending positions of this resulting interval are respectively the smallest starting position and largest ending position of this group of intervals. The **coalesce** operator can be used in the FROM clause with the following syntax:



where T is the input track (the individual enhancers), and the optional “**with** <*vd*>**using** <value-model >” part is for deriving the value of each resulting interval based on the mathematical operation <*vd*> and value model <value-model >. STQL has a highly flexible design for value derivation that distinguishes itself from other existing languages, the details of which will be discussed shortly. The output of this operation is a new track that contains the merged intervals. An illustration of the **coalesce** operator is given in Fig. 1. Complete query examples using **coalesce** and other advanced constructs will be given later.

**Fig. 2:**
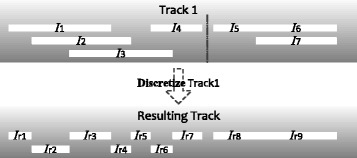
An example that illustrates the discretize operator. I_1_..I_7_ are intervals in the input track that are allowed to overlap with each other, while I_r1_..I_r9_ are non-overlapping intervals in the resulting track after the discretization operation. Each resulting interval is defined by the boundary positions of some input intervals. For example, I_r1_’s starting position is the same as I_1_’s starting position, and its ending position is equal to I_2_’s starting position minus one. It should be noted that the output intervals I_r8_ and I_r9_ are adjacent to each other since they were produced from input intervals that were also adjacent (I_5_ and I_6_/I_7_)



Another common operation for processing overlapping regions is to use their boundary locations to define discrete intervals that can be adjacent to each other (Fig. 2). This is useful when the next analysis step requires all intervals to be non-overlapping, for example when each genomic location should be classified as either within an interval (such as a protein binding site) or not. In STQL, this type of operations can be performed by using the **discretize** operator in the FROM clause:

#### Creating a new track from two existing tracks

The FROM and WHERE clauses together allow for some basic joins of multiple signal tracks. To make more advanced types of track joins easy to perform, STQL provides convenient constructs for them.

**Fig. 3:**
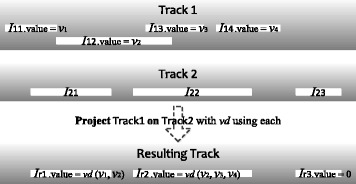
An example that illustrates the project on operator. I_11_..I_14_ are intervals in input track 1, I_21_.. I_23_ are intervals in input track 2, while I_r1_..I_r3_ are intervals in the resulting track after the projection. The locations of the intervals in the resulting track are directly from the intervals in input track 2. The value of I_r1_ is determined by the values of I_11_ and I_12_ since they are the ones that overlap with I_21_. The exact way of computing the value depends on the mathematical operator and the value model (which we use vd(*v*_1_, *v*_2_) here to mean the computation based on the values from intervals I_11_ and I_12_). Similarly, the value of I_r2_ is determined by the values of I_12_, I_13_ and I_14_ since they are the intervals that overlap with I_22_. Since I_23_ does not overlap with any intervals in track 1, it does not receive any value from track 1 but is instead given the default value of 0



In the first type of advanced track joins, a track T_2_ defines the positional information of the resulting intervals and another track T_1_ defines their values (Fig. 3). This is most typically used when T_2_ corresponds to gene annotations, T_1_ is a signal track of experimental values, and the goal is to compute an aggregated signal value for each gene based on the experimental data. In STQL, this type of operations is described as projecting T_1_ on T_2_ in the FROM clause:

where the optional “**metadata**” part is for specifying whether non-default attributes of the input intervals are to be inherited by the resulting intervals, which will be explained later.

It is often useful to partition the whole genome into bins of a fixed size, and compute an aggregated signal value for each bin. By choosing a suitable bin size, the signals are smoothed locally and some downstream tasks can be carried out more efficiently due to the reduced data resolution and easily computable bin locations. This binning operation can be performed in STQL by projecting a signal track on a bin track dynamically created using the **generate bins with length** construct in the FROM clause:



where <bin-size > is the size of each bin in base pairs. The output intervals are adjacent bins of this size covering the whole genome.

Two different signal tracks are usually compared to find out genomic locations covered by both tracks, one track but not the other, or either track. STQL supports these operations by the **intersectjoin**, **exclusivejoin** and UNION ALL constructs.

**Fig. 4:**
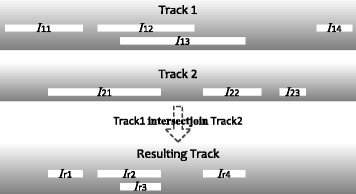
An example that illustrates the intersectjoin operator. I_11_.. I_14_ are intervals in input track 1, I_21_.. I_23_ are intervals in input track 2, while I_r1_..I_r4_ are intervals in the resulting track after the intersect-join. I_11_ and I_21_ each produces only one resulting interval (I_r1_ and I_r2_ respectively) because they only overlap with I_21_. I_13_ produces two resulting intervals (I_r3_ and I_r4_) because it overlaps with both I_21_ and I_22_. I_14_ does not produce any resulting interval because it does not overlap with any intervals in track 2



**Fig. 5:**
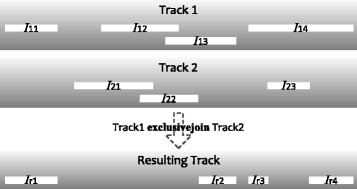
An example that illustrates the exclusivejoin operator. I_11_.. I_14_ are intervals in input track 1, I_21_.. I_23_ are intervals in input track 2, while I_r1_..I_r4_ are intervals in the resulting track after the exclusive-join. The whole interval of I_11_ remains to become I_r1_ in the resulting track, because it does not overlap with any interval in track 2. In contrast, the whole interval of I_12_ is not included in the resulting track, because it is completely covered by I_21_ and I_22_. For I_13_, the part of it not covered by I_22_ becomes interval I_r2_ in the resulting track. Finally, I_14_ is being cut by I_23_ into two intervals I_r3_ and I_r4_ in the resulting track



**intersectjoin** considers every pair of overlapping intervals from the two input tracks, and takes their intersection as a resulting interval (Fig. 4). It can be used in the FROM clause:

**exclusivejoin** considers every interval from the first input track, and removes all parts of it that overlap any intervals in the second input track (Fig. 5):

Finally, UNION ALL forms a new track that keeps all intervals from the two input tracks without removing duplicates. The tracks involved must have the same schema. It can be used to join the resulting tracks of two queries. Since the result of UNION ALL is also a signal track, it can be repeatedly applied to join the resulting track with another signal track. For example, the following query takes the union of three signal tracks to form a new track (where the alias NtInt stands for “new track interval”):



#### Value derivation and inheritance of metadata

All advanced constructs described above allow the derivation of values for the resulting intervals. Having a flexible way to manipulate interval values is crucial to many types of analysis. In STQL, two value models are used for interpreting and deriving signal values. In the **EACH MODEL**, each genomic location within an interval is considered to individually own the signal value of the interval. For example, if signal values represent normalized read counts, an interval having a certain value means that every genomic location in the interval is covered by that number of reads on average. On the other hand, in the **TOTAL MODEL**, all genomic locations within an interval is considered to collectively own the signal value of the interval. For example, if the signal value indicates the total number of MBDCap-seq [18] reads aligned to an interval, all genomic locations of the interval collectively own the signal value. The following query projects these intervals onto 100bp bins, and computes the raw signal of each bin based on the number of bases overlapping with the bin as a fraction of the interval length:



For STQL operations that involve the creation of intervals described above, the value of each resulting interval is determined by the specified value model and mathematical operation. In general, the value of each resulting interval is derived in three steps:

1. For each interval in the input tracks, the signal value at each of its genomic locations is determined.
2. For each interval in the resulting track, the signal value at each of its genomic locations is computed based on the values at the same location of the input intervals computed in Step 1.
3. For each interval in the resulting track, a final value is computed by aggregating the values of its genomic locations computed in Step 2.

For Step 1, if the **EACH MODEL** is used, the value at each genomic location is simply the value of the corresponding interval. On the other hand, if the **TOTAL MODEL** is used, each genomic location is given an equal share of the value of the interval.

Step 2 depends on the exact STQL operation being performed, the details of which will be explained next.

Step 3 computes the average over all values of the genomic locations within the resulting interval.

For example, suppose in Fig. 4 every interval in the two input tracks has value 1, and the two tracks are joined using the **intersectjoin** construct with the **vd_sum** operation, which adds up values from different intervals location by location in Step 2 of value derivation. If the **EACH MODEL** is used, the values of *I*_*r*1_, *I*_*r*2_, *I*_*r*3_ and *I*_*r*4_ will all be 2. This is because in Step 1, every genomic location of the input intervals receives a value of 1; In step 2, every genomic location of the resulting intervals is given a value of 1+1=2; In Step 3, since every location in each resulting interval has the same value, taking the average will give the same value of 2.

On the other hand, if the **TOTAL MODEL** is used, the values of the resulting intervals will depend on the lengths of the intervals. For example, the value of *I*_*r*1_ will be *I*_11_.value/**length**(*I*_11_) + *I*_21_.value/**length**(*I*_21_), since the two fractional values are respectively given to each genomic location of *I*_11_ and *I*_21_ in Step 1, and Steps 2 and 3 are similar to the case for the **EACH MODEL**.

Table 2 shows the full list of mathematical operations in STQL and how the value of each genomic location of the resulting interval is computed in Step 2. The operations provided include 1) arithmetic operations (summation, averaging, subtraction, multiplication and division), 2) maximum and minimum function, and 3) direct copying of values from the interval from input track 1 or track 2.

**Table 2:** The full list of mathematical operations defined for STQL operations that create intervals

STQL operation	**Coalesce**, **discretize**	**Intersectjoin**	**Exclusivejoin**
	or **project on**		
Values involved	*v*_1_...*v*_*n*_	*v*_1_, *v*_2_	*v*_1_
**vd_sum**	$\sum _{i=1}^{n} v_{i}$	*v*_1_+*v*_2_	N/A
**vd_avg**	$\frac {\sum _{i=1}^{n} v_{i}}{n}$	(*v*_1_+*v*_2_)/2	N/A
**vd_diff**	N/A	*v*_1_−*v*_2_	N/A
**vd_product**	$\prod _{i=1}^{n} v_{i}$	*v*_1_×*v*_2_	N/A
**vd_quotient**	N/A	*v*_1_÷*v*_2_	N/A
**vd_max**	$\max _{i=1}^{n} v_{i}$	max(*v*_1_,*v*_2_)	N/A
**vd_min**	$\min _{i=1}^{n} v_{i}$	min(*v*_1_,*v*_2_)	N/A
**vd_left**	N/A	*v*_1_	*v*_1_
**vd_right**	N/A	*v*_2_	N/A

Starting from the third row, the first column shows the names of these mathematical operations that can be used in the <*vd* > placeholders in statements involving **coalesce**, **discretize**, **project**, **intersectjoin** and **exclusivejoin**. These mathematical operations are used in Step 2 of value derivation. The second row defines the values involved in the operations. In the case of **intersectjoin**, exactly two values are involved, namely *v*_1_ from the first track and *v*_2_ from the second track. In the case of **exclusivejoin**, exactly one value is involved, namely *v*_1_ from the first track. In the case of **coalesce**, **discretize** and **project**, all values come from the same track and there can be one or more values involved. N/A indicates mathematical operators that cannot be used with the STQL operations

For **intersectjoin**, each resulting interval is formed by exactly two intervals one from each input track, and thus all nine types of operation are well-defined. For **exclusivejoin**, each resulting interval is formed by one interval from the first input track and zero, one or more intervals from the second track. Only the unary operator **vd_left** is applicable. For **coalesce** and **discretize**, only one track is involved, while for **project**, all values come from track 1. For these three constructs, each resulting interval can be formed by one, two or more than two input intervals. Without a defined order of these intervals, the **vd_diff**, **vd_quotient**, **vd_left** and **vd_right** operations cannot be defined and are thus not allowed.

If the value model and mathematical operation are not specified, the resulting intervals will be given the value NULL.

Each interval may contain additional attributes that are called metadata, such as the name of a gene and the confidence score of a signal peak. For some of the interval-creating constructs, these metadata can be inherited from the input intervals to the resulting intervals using “**metadata**”. For **project**, the metadata are inherited from the input intervals in the second track, the track that defines the positional information of the resulting intervals. For **intersectjoin** and **exclusivejoin**, the metadata are inherited from input intervals in the first track.

#### Using dynamically created tracks

In the FROM clause, in addition to using existing tracks in the database, one could also create new tracks dynamically using either a nested query or one of the above track operations. For example, the following query first takes the **intersectjoin** of two tracks, and then selects out the resulting intervals with a value larger than 2:



An alias is given to the intervals of the dynamically created track, which can then be referred to in the SELECT and WHERE clauses.

#### Data definition and manipulation statements

STQL also contains statements for creating and deleting signal tracks, and loading data into a signal track from a local file.

The CREATE TRACK statement is used to create a new track and add it to the database. It has two different forms:



In the first form, a new empty track is created with the name specified at the placeholder <track-name >. The list of attributes and their data types (string, int or float) are then listed within the brackets. In the second form, an STQL query is executed and the result is stored as a new track with the name specified at <track-name >. If the query results do not form a valid signal track, i.e., it does not have all the required attributes for a signal track, an error will be produced when a query tries to use the query results as a track. This second form of CREATE TRACK is particularly useful when multiple STQL statements are submitted in the same block on the START Web site, where the intermediate results produced by a step are stored in a temporary signal track using a CREATE TRACK statement, which can then be accessed by the queries in the subsequent steps.

Conceptually, tracks created by a CREATE TRACK statements persist in the database, but the ones created through the START Web interface (described below) are automatically removed after a certain amount of time to control the space used by each user.

The DROP TRACK statement deletes a track in the database:



Execution of this statement requires the user to have the corresponding permission. There are other security measures in STQL that will be explained when we describe START in detail. The DROP TRACK statement is commonly used to remove intermediate tracks created by the CREATE TRACK statement that are no longer needed.

STQL also allows loading data into a track by using theLOAD DATA LOCAL INPATH INTO TRACK statement, for example after a new track is created using the first form of the CREATE TRACK statement:



where <file-path > is the path of the data file, <track-name > is the name of the track into which the data are to be loaded, and the OVERWRITE option is for specifying whether any existing data in the track are to be removed.

#### Selection and looping over signal tracks

A final feature of STQL, which is very useful when analyzing a large number of signal tracks, is selecting tracks based on their attributes, and looping over the selected tracks for repeating some operations. This feature is provided by the FOR TRACK IN () statement with two forms:



In both forms, <track-variable > is a variable for the intervals of a selected track in the STQL query, <track-category > is the category of signal tracks to be selected, <track-selection-conditions > states extra conditions for track selection, <STQL-query > is the query to be performed on each selected track, and <output-track-name > is the name of the track to store the results.

Specifically, <track-selection-conditions > is a list of attribute names and values delimited by “and”. For example, if one wants to select all ChIP-seq binding peaks in the GM12878 cell line produced by the ENCODE Stanford/Yale/Davis/Harvard (SYDH) sub-group, and stores the union of all these peaks into an output signal track, the following statement can be used:



In this statement, a track is selected if it belongs to the ENCODE SYDH transcription factor binding sites (SYDH TFBS) category, contains data from GM12878 cells, and has “Pk” (peak) as part of its track name. The “LIKE” syntax of SQL for string matching with wildcards can be used in specifying track selection conditions. For each selected track, its intervals are represented by the variable TInt, and the union of the intervals from these tracks are stored in the output track “AllPeaks”.

As shown in this example, the first form of the FOR TRACK IN () statement combines the results from all the selected tracks by a UNION ALL operation. The second form, on the other hand, allows the query result from each selected track to be stored in a separate output track name <output-track-name > concatenated with the name of the selected track), which can then be post-processed by using other STQL queries. Currently STQL does not support nested FOR TRACK IN () statements.

To demonstrate the use of STQL, in Additional file 1 we provide 14 sample queries, including both simple and complex ones.

### Signal Track Analytical Research Tool (START)

**Fig. 6:**
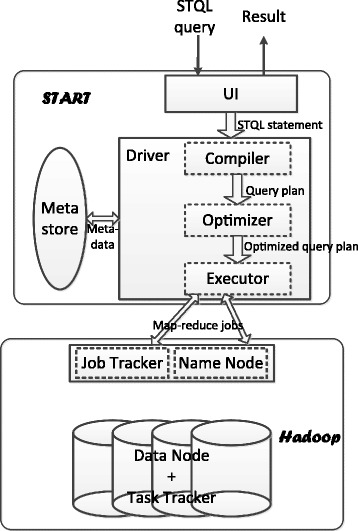
The overall architecture of START. The Web-based user interface helps users select signal tracks, construct STQL queries, submit queries, and retrieve execution results. It also provides various additional functionality, such as user management, storage for queries, data files and result files, and sharing of queries with other users. The metastore provides information about the stored signal tracks in the backend database. When a query is sent to the backend system, it is handled by a driver that consists of three main components. First, a compiler checks for potential syntactic and permission errors, and produces a parse tree of the query if no errors are found. Second, an optimizer analyzes the parse tree and determines an execution plan optimized for efficiency. Third, an executor calls the underlying system to execute the query. The underlying system is based on the Hadoop framework, which distributes the data files needed and performs the actual computations on multiple machines in parallel. When a job is finished, the results are stored and the user is notified to preview or download them using the user inferface

We developed a system called Signal Track Analytical Research Tool (START) for running STQL queries on multiple machines in parallel. START involves a front-end Web-based user interface and a backend execution system (Fig. 6). The purpose of the Web-based user interface is to provide a simple way for users to test out STQL. We have pre-loaded around 10,000 signal tracks from ENCODE, Roadmap Epigenomics, FANTOM5 [9] and other sources into our database for users to integrate these data into their analyses. In additional to the standard file formats supported by START, we also imported some commonly used data in other formats (such as gene annotation in.gtf format) using our custom scripts.

We encourage users who want to use STQL to analyze large amounts of private data to install START locally on their own machines. We provide an installation package at https://github.com/stql/start/wiki/Install-START-in-your-own-cluster. START can be run on either a single machine or a cluster of machines. All source code of START can be found at https://github.com/stql/start, distributed under Apache License v2.0.

#### Front-end: Web-based user interface

**Fig. 7:**
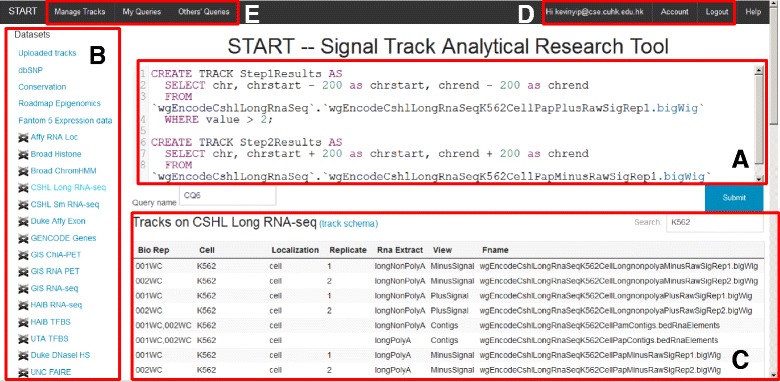
The user interface of START. **a** The main text box for entering STQL queries. **b** A list of signal track categories of the tracks stored in the backend database. **c** The list of signal tracks in the selected category. **d** Menu items related to user accounts. **e** Menu items for managing and sharing stored queries and files

START provides a Web-based user interface at http://yiplab.cse.cuhk.edu.hk/start/ (Fig. 7). It provides a main input box for entering STQL queries. Multiple queries can be entered at the same time, in which case each query should store its results in a temporary track, and the results of the last query will be returned by the system as the final results.

Four features are provided to help users construct their queries. First, a user can use his/her previous queries or queries shared by other users as template to perform new analyses by changing only the parts that differ. Second, signal tracks stored in the backend database are listed in categories. A user can select signal tracks using the built-in searching function based on text matches in all track attributes. The names and data types of the attributes of the intervals in a signal track can be shown by clicking the “track schema” link. Third, in the main input box, STQL keywords are highlighted in different colors to help users spot syntax errors. Finally, an extensive help system is provided on the START Web site with detailed documentations and example queries.

A user can use all the functions described above and submit STQL queries with or without logging in. Users logged in (after a free registration) can additionally store their own executed queries, data files, and query results on START. Each user is given a different database name such that files of different users are completely separated. Data files can be uploaded in a number of standard file formats (.bed,.bedGraph and.wig), and multiple files can be uploaded at the same time in a zip package. All the supported formats have the chr, chrstart and chrend attributes defined. The value attribute is defined in.bedGraph and.wig, while for the.bed format it is left as NULL. The schema of the uploaded data is automatically generated based on this mapping. A user can also share or unshare queries with other users. START ensures that only queries explicitly shared by the owner can be seen by other users, and data files uploaded by a user cannot be accessed by other users.

A user submits a query by entering a name of the query and pressing the “Submit” button. A checker module at the backend is then invoked immediately. If any syntax error or permission problem is detected, the query is rejected and an error message is returned to the user without executing the query. Otherwise, a query job will be created at the backend and the actual processing of it will be carried out when the execution system becomes available.

When a query has been executed, the user can preview the first few rows of the results on START, or download all the results in a file. Users are not required to wait for a query to complete by keeping the browser open, because when a user returns to the START Web site, he/she can find all executed queries from the menu and the result files can be downloaded from the corresponding page linked from the list of executed queries for recently executed queries.

#### Back-end: parallel-execution system

In the back-end of START, STQL queries are translated into optimized executable programs that are run on a cluster of machines in parallel. The parallelization is powered by the Hadoop [19] distributed data storage and MapReduce framework for big data processing. Intervals on each chromosome are mapped to the same computing node. The translation of STQL into executable programs is assisted by Hive [20], a warehousing infrastructure built on top of MapReduce. It provides an SQL-like query language called HiveQL, and it translates HiveQL queries into Hadoop programs. We used Hive to execute parts of STQL queries that can be directly translated into HiveQL queries, and handled some signal track-specific constructs (**is closest to each**, **intersectjoin**, **exclusivejoin**, **project**, **coalesce** and **discretize**) by our own programs. An advantage of Hive is that it can work on raw data files directly, without requiring a long processing time of converting the raw data files into a particular format before the corresponding tracks can be used in the queries. This feature makes it very efficient for users to use their own signal tracks in the queries.

#### Interface between front-end and back-end: Metastore

In order for the front-end user interface to obtain information about the stored signal tracks in the database, it has to obtain the information from the back-end. The metastore provides such information and acts as an interface between the front-end and back-end systems. The metastore records three main types of information, namely 1) the schema of each signal track, i.e., the exact names and data types of the attributes of the intervals in each signal track, 2) the physical locations of the corresponding data files in the backend system, which is stored in a Hadoop file system (HDFS), and 3) the organization of the signal tracks into categories, and the attributes of the signal tracks in each category. When any of these three types of information is updated at the back-end, the Web-based user interface always displays the most updated information by retrieving it from the metastore in real time.

#### Back-end: translation

At the back-end, we use Hadoop [19] for distributed data storage, which includes a MapReduce framework for big data processing. High-level STQL queries are translated into executable programs (MapReduce jobs) that can be executed by Hadoop. This translation is facilitated by Hive [20], a warehousing infrastructure built on top of MapReduce. It provides an SQL-like query language called HiveQL, and it translates HiveQL queries into Hadoop programs. We extended HiveQL to include syntactic constructs specific to STQL. An advantage of Hive is that it can work on raw data files directly, without requiring a long processing time of converting the raw data files into a particular format before the corresponding tracks can be used in the queries. This feature makes it very efficient for users to use their own signal tracks in the queries.

To execute an STQL query, the first step is to translate it to a sequence of operations. It involves four sub-steps, namely 1) parsing the STQL statement and producing an abstract syntax tree (AST), 2) traversing the AST to create a query block (QB) and record necessary parsing information in the QB, 3) interacting with the metastore to retrieve metadata of the involved signal tracks, and 4) generating a query plan in the form of a directed acyclic graph (DAG) of logical operations based on the QB.

#### Back-end: execution

**Fig. 8:**
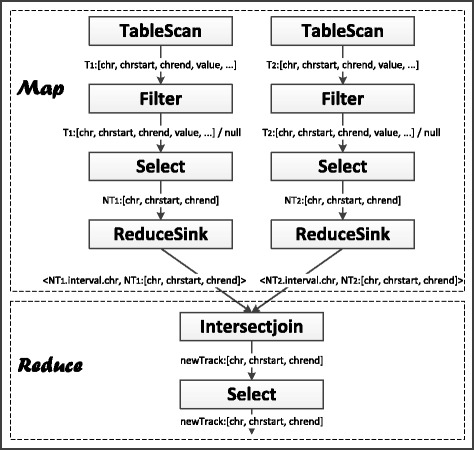
A typical MapReduce job created by the executor from an STQL query

The DAG of logical operations are then converted into executable jobs in Hadoop. Figure 8 shows a simple example illustrating the typical steps in such a MapReduce job. In the Map phase, the TableScan operator fetches one interval from a signal track at a time, and forwards all attributes of the interval to the Filter operator. Upon receiving an interval, the Filter operator judges whether the interval satisfies the predicate in the WHERE clause. If the predicate holds true for the interval, the Filter operator forwards the interval to the Select Operator. The Select operator selects the attributes of the interval necessary for the calculations. It then forwards the results to the ReduceSink operator, which creates a key-value pair for the interval it receives. This finishes the Map phase. Based on the keys, the intervals are sent to different machine nodes for further processing.

In the Reduce phase, the Intersectjoin operator maintains buffers for caching the intervals it receives. When all intervals have been received, it proceeds with the actual computations. Whenever a resulting interval is produced, it forwards the interval to the Select operator, which supplies all attributes that need to be returned in the final outputs.

#### Back-end: optimization

Together, the compiler and executor described above are sufficient for turning STQL statements into executable programs. However, the straight-forward way of translating the queries into executable programs could make the programs inefficient. The goal of the optimizer is to find ways to perform the queries more efficiently.

The optimizer makes use of several key ideas. First, it removes interval attributes that are not needed as early as possible, to reduce the amount of data transfer between computing nodes. Second, when a join is performed between two tracks, instead of producing the Cartesian product, the optimizer tries to use more efficient algorithms to reduce both the computation and the amount of intermediate results. For example, by pre-sorting both signal tracks involved, sometimes it is possible to perform a single linear scan of the resulting sorted tracks to produce the join result. Finally, if the **generate bins with length** construct is used, instead of creating the actual bins, the optimizer computes the overlapping bins of each interval, so that projection can be done efficiently without considering the bins that do not overlap any intervals.

### STQL grammar rules

In the “Results” section we have explained the syntax of STQL using high-level terms and examples. Here we present the complete set of grammar rules that define STQL.

STQL_STATEMENT := DDL | DML | QUERY

DDL := CREATE_TRACK | CTAS | DROP_TRACK

CREATE_TRACK := create track TRACKALIAS*LBracket* SCHEMA *RBracket*

CTAS := create track TRACKALIAS as REG_QUERY

SCHEMA := ATTRNAME DATA_TYPE (, ATTRNAME DATA_TYPE)*

DROP_TRACK := drop track TRACKALIAS

DML := LOAD_DATA

LOAD_DATA := load data local inpath *Filepath* (overwrite)? into track TRACKALIAS

DATA_TYPE := string | int | float

QUERY := REG_QUERY | FOR_LOOP

REG_QUERY := SELECT_STAT FROM_STAT (WHERE_STAT)? (GROUPBY_STAT)? (ORDERBY_STAT)?

FOR_LOOP := for track TRACK_VAR in *LBracket* *TrackProperty* *RBracket* (REG_QUERY combined with UNION as TRACKALIAS | CTAS)

TRACK_VAR := *Identifier*

FROM_STAT := from FROM_SOURCE

FROM_SOURCE := MULTIPLETRACK

TRACK := RAW_TRACK | TRANSFORM_RES | OVERLAPJOIN_RES | SUBQUERY | UNION_RES

MULTIPLETRACK := TRACK (, TRACK)*

UNION_RES := *LBracket* TRACK UNION TRACK (UNION TRACK)* *RBracket* TRACKALIAS

UNION := union all

RAW_TRACK := (CATEGORY.)?TRACKNAME ((as)? TRACKALIAS)?

CATEGORY := *Identifier*

TRACKALIAS := *Identifier*

TRANSFORM_RES := TRANSFORM_OP |*LBracket* TRANSFORM_OP *RBracket* TRACKALIAS

TRANSFORM_OP := TRANSFORM (with VALUE_DER)?

TRANSFROM := COALESCE TRACK | DISCRETIZE TRACK

COALESCE := coalesce

DISCRETIZE := discretize

OVERLAPJOIN_RES := OVERLAPJOIN_OP |*LBracket* OVERLAPJOIN_OP *RBracket* TRACKALIAS

OVERLAPJOIN_OP := OVERLAPJOIN (with (VALUE_DER_METADATA | VALUE_DER | META_DATA))?

OVERLAPJOIN := INTERSECTJOIN | EXCLUSIVEJOIN | PROJECT

INTERSECTJOIN := TRACK intersectjoin TRACK

EXCLUSIVEJOIN := TRACK exclusivejoin TRACK

PROJECT := project TRACK on (TRACK | CREATE_BINS)

CREATE_BINS := generate bins with length *Integer*

VALUE_DER_METADATA := VALUE_DER, META_DATA | META_DATA, VALUE_DER

VALUE_DER := VD_TYPE using VALUE_MODEL

VD_TYPE := vd_sum | vd_diff | vd_product | vd_quotient | vd_avg | vd_max | vd_min | vd_left | vd_right

META_DATA := metadata

VALUE_MODEL := VM_TYPE model

VM_TYPE := each | all

SELECT_STAT := select ((distinct)? FIELD (, FIELD)* | SELALLEXP)

SELEXP := FIELD (as ATTRNAME)?

SELALLEXP := *

FIELD := ARITH_FUNC | AGG

ARITH_FUNC := (MUL_DIV |*Number*) ((+ |−) (MUL_DIV |*Number*))?

MUL_DIV := (ELEM |*Number*) ((* | /) (ELEM |*Number*))?

ELEM := INTERVAL_ATTR |*LBracket* ARITH_FUNC *RBracket*

INTERVAL_ATTR := ATTRNAME | TRACKNAME.ATTRNAME

TRACKNAME := *Identifier* | TRACKALIAS

ATTRNAME := chr | chrstart | chrend | value |*Identifier*

AGG := AGG_FUNC *LBracket* INTERVAL_ATTR *RBracket* | COUNT_ALL

AGG_FUNC := count | max | min | avg | sum

COUNT_ALL := count *LBracket* SELALLEXP *RBracket*

WHERE_STAT := where (OR_PREDICATE | CLOSEST_PREDICATE)

OR_PREDICATE := AND_PREDICATE (or AND_PREDICATE)?

AND_PREDICATE := NOT_PREDICATE (and NOT_PREDICATE)?

NOT_PREDICATE := PREDICATE | not (PREDICATE |*LBracket* OR_PREDICATE *RBracket*)

PREDICATE := NUMERIC_COMP | LOCATION_COMP | PATTERN_MATCHING

NUMERIC_COMP := (INTERVAL_ATTR | INTERVAL_LENGTH | INTERVAL_DIS |*Number*) COMP_OP (INTERVAL_ATTR | INTERVAL_LENGTH | INTERVAL_DIS |*Number*)

INTERVAL_LENGTH := length *LBracket* (TRACKNAME | CONS_INTERVAL) *RBracket*

INTERVAL_DIS := distance *LBracket* (TRACKNAME | CONS_INTERVAL), (TRACKNAME | CONS_INTERVAL) *RBracket*

COMP_OP := < |=|!=|>|<=|>=

LOCATION_COMP := (TRACKNAME | CONS_INTERVAL) LOC_COMP_OP (TRACKNAME | CONS_INTERVAL)

LOC_COMP_OP := overlaps with | precedes | follows | coincides with | is prefix of | is suffix of | is adjacent to | is within | contains | is upstream of | is downstream of

CONS_INTERVAL := *LeftSquareBracket* CHR, CHRSTART, CHREND (, STRAND)? *RightSquareBracket*

CHR := *Identifier*

CHRSTART := *Integer*

CHREND := *Integer*

STRAND := + |−

PATTERN_MATCHING := INTERVAL_ATTR (not)? like *RegularExpression*

CLOSEST_PREDICATE := TRACKNAME is closest to each TRACKNAME

GROUPBY_STAT := group by INTERVAL_ATTR (, INTERVAL_ATTR)*

ORDERBY_STAT := order by INTERVAL_ATTR (, INTERVAL_ATTR)*

SUBQUERY := *LBracket* QUERY *RBracket* TRACKALIAS

## Results

### Comparison with other approaches

To evaluate the simplicity of STQL and the correctness and efficiency of START in executing STQL queries, we compared STQL with three other approaches in performing the same analysis tasks.

First, we used the Web-based user interface to submit the 14 example STQL queries to START, and downloaded the resulting output files. For each query, we measured the time required, from submitting the query to getting the final result file. We also used bedtools [6], Galaxy [4] and custom Python scripts to perform the same tasks. We then checked if the output files produced by the different approaches were the same, and compared the time required.

The source code of these three implementations is available at https://github.com/stql/start/wiki/Website-User-Manual#source-code-for-other-tools. For some queries, we were unable to find a trivial way to perform exactly the same operations using one or more of these approaches. We note that this does not mean it is impossible to carry out the corresponding analyses using these approaches, but the solutions could be non-trivial. On the other hand, it was fairly easy to write STQL queries to perform the tasks, and the STQL queries involved fewer tokens than both the bedtools and Python scripts for all the 14 tasks (Table 3).

**Table 3:** Number of tokens involved in the code of the different approaches on the 14 example queries

Query	START	Bedtools	Python
SQ1	21	63	158
SQ2	30	71	220
SQ3	6	26	202
SQ4	34	61	336
SQ5	23	28	162
SQ6	12	24	146
SQ7	13	25	117
SQ8	14	25	91
CQ1	38	N/A	288
CQ2	53	N/A	460
CQ3	102	N/A	471
CQ4	105	164	500
CQ5	266	N/A	462
CQ6	50	83	202

N/A indicates cases in which we were unable to find a trivial way to perform the analysis using the approach

Based on the execution results, START was able to produce identical output files as those produced by the Python scripts for all 14 queries. In some cases, bedtools and Galaxy produced results different from STQL. For example, for SQ5, bedtools could produce the same intervals as STQL but could not derive the required values. In general, STQL was found to be very expressive, and its value derivation capability was particularly flexible.

Table 4 shows the execution time of the different approaches. For START, we used a Hadoop cluster to execute the queries. The cluster contained 22 machines, each with an Intel Core i7-3770 CPU at 3.40 GHz, 16 GB main memory, and disks with I/O speed of 133.75 MB/s. For bedtools and python scripts, we used a single machine to execute the queries, with an Intel Core i7-3770 CPU at 3.40 GHz, 16 GB main memory, and disks with I/O speed of 156 MB/s. For Galaxy, we used its online version (https://usegalaxy.org/). Since the hardware used for each approach was different, it is not meaningful to use the measured time to argue which approach is more efficient. Instead, the main purpose of this time comparison is threefold. First, it shows that for some of the tasks that STQL could easily handle, we could not find a way to perform the same tasks using bedtools or Galaxy (marked as N/A in Table 4), suggesting that it is more difficult or even impossible to perform these tasks using these tools. Second, in general, START could finish each task within reasonable time even without using algorithms and data structures specially designed for each task as we did with the Python scripts. Third, when the data files were large, the implicit parallel execution of START made it easy to speed up the analysis, without requiring the user to write anything about parallelization in the STQL queries. For example, in SQ1 and SQ2, the data files involved were larger than 1 GB, and START was able to finish the task faster than the other approaches due to its parallel computations.

**Table 4:** Execution time of the different approaches on the 14 sample queries in seconds

Query	Number of input tracks	Number of input intervals	Number of output intervals	START	Bedtools	Python	Galaxy
SQ1	1	57,059,743	23,857,046	207	407	1171	N/A
SQ2	2	11,517,945	33,312	50	135	184	N/A
SQ3	2	51,417	14,026	39	0.04	0.3	23
SQ4	2	11,517,945	1,054,854	47	21	42	408
SQ5	1	8,898,501	450,380	52	7	125	270
SQ6	2	18,839	5702	46	0.04	21	N/A
SQ7	1	2,619,444	36,366	31	6	5	44
SQ8	1	2,619,444	1	33	2	3	30
CQ1	52	1,514,863	2,590,502	86	N/A	36	N/A
CQ2	3	2,938,174	29,225	300	N/A	7	N/A
CQ3	53	4,134,307	76,041	1340	N/A	84	N/A
CQ4	100	32,297,907	68,031	1680	262	420	N/A
CQ5	5	257,369,824	264	360	N/A	5289	N/A
CQ6	2	65,412,859	4,006,220	119	207	483	N/A

N/A indicates cases in which we were unable to find a trivial way to perform the analysis using the approach

We have also compared STQL with SQL, and found that some operations are much more difficult to perform using SQL than STQL. The details are provided in Additional file 1.

### Case study

To test if STQL is easy to learn and to use, we asked one of us (KH-OY), who was trained as a biologist and had received minimal formal training in computer programming (including SQL), to analyze some sequencing data using two different approaches. The data involved were DNA methylation data we produced by MBDCap-seq [18] on 60 pairs of human hepatocellular carcinoma (HCC) tumor and matched non-tumor tissues. The goal was to compute DNA methylation levels at gene promoters, and identify promoters with significant differential methylation between the tumor and non-tumor groups.

The first analysis approach was to implement the analysis pipeline by writing custom Perl scripts. The second approach was to write STQL queries and submit them through the START Web interface, to perform exactly the same analysis.

Specifically, for each protein-coding gene in Gencode [21] v19, the promoter region was defined as the +/-500bp around the transcription start site. The average methylation signal at each promoter was computed separately for the tumor and non-tumor samples. Finally, the full list of genes and their promoter differential methylation fold change values were reported. The Perl scripts and the STQL queries written, as well as the resulting output files, are all available at https://github.com/stql/start/raw/master/for-download/STQL_HCC_Diff_Methyl_files.zip.

The STQL queries are found to be simpler than the Perl scripts. For instance, the Perl scripts involve 253 lines of code in total, while the STQL queries involve only 55 lines.

The two approaches led to identical results. Among the top five most hyper-methylated promoters, FGF19 is related to HCC tumor promotion [22], FGF4 is related to HCC drug response [23], and HLX is involved in normal liver development [24]. Although the other two genes have yet to link with HCC, their roles in cancer development have been reported. MYEOV deregulation contributes to malignant transformation of different cell types [25], while LRR1 is involved in cell growth control [26]. These results suggest that it is indeed fairly easy for someone without very strong computer science background to learn and use STQL to produce biologically meaningful results.

## Discussion

The main purpose of START is to demonstrate the use of STQL. If it is to be used for routine large-scale data analysis, the signal tracks stored in its database should be frequently updated. To achieve that, we are exploring the possibility to hook up START with major genome databases for automatic data updates, or to setup a local copy of START at these sites.

Currently START supports only signal tracks based on the hg19 human reference genome. Conceptually, STQL can also support other versions of the human reference genome, as well as other species. They will be supported in future versions of START.

In the current implementation of the track operators, parallelization is achieved by sending all intervals on one chromosome to one computing node, which is not very efficient due to the very different sizes of the human chromosomes. We are developing new algorithms to provide sub-chromosome level parallelization [27].

There are other emerging distributed computing frameworks. For instance, Spark [28] is a successor of Hadoop that keeps data files in memory such that a user issuing multiple queries on the same data files could enjoy significant speed up. We will explore the possibility of using Spark as the underlying framework to further improve the efficiency of START.

Since in most applications joining of different tracks does not involve pairing of intervals from different chromosomes, by default STQL only considers pairing of intervals from the same chromosome to avoid the unnecessary computational overhead. If it is necessary to pair intervals from different chromosomes, one way is to save chromosome names in a new attribute and replace the chr attribute by a common fake chromosome name before the join operation. After the join, the actual chromosome names can be copied back. We will consider adding an operation that allows across-chromosome comparisons if many applications find it useful.

## Conclusions

In this paper, we have described the Signal Track Query Language (STQL), an SQL-like declarative language that allows users to perform a variety of analysis by specifying only the analysis goals rather than all the computational details. We have demonstrated some typical use of STQL through 14 example queries, which cover both simple and composite analysis tasks. We have used these example queries to show that STQL usually provides a simpler solution than several other popular analysis approaches.

To make it easy to write and execute STQL queries, we have developed the Signal Track Analytical Research Tool (START). The Web-based user interface of START allows simple integrated analysis of private and commonly-used public signal tracks. It also provides the management of stored data and queries. The back-end system of START automatically translates STQL queries into executable programs that are run in parallel on multiple machines, without requiring the analysts to diverge their attention to finding a suitable parallelization strategy.

Together, STQL and START provide a simple and generic way for analyzing a large number of genomic signal tracks.

## Availability and requirements

**Project name:** Signal Track Analytical Research Tool**Project home page:** https://github.com/stql/start**Operating system:** Linux (Ubuntu recommended)**Programming language:** Java**Other requirements:** JDK 6 or higher, Hadoop installation**License:** Apache License v2.0**Any restrictions to use by non-academics:** license needed

## Additional file

Additional file 1 Supplementary Materials. (PDF 198 kb)

## Abbreviations

AST: Abstract syntax tree
ChIP-seq: Chromatin immunoprecipitation followed by high-throughput sequencing
DAG: Directed acyclic graph
ENCODE: Encyclopedia of DNA elements
eRNA: Enhancer RNA
FANTOM5: Functional annotation of The Mammalian Genome Phase 5
HDFS: Hadoop file system
HOT: High occupancy of trancription-related factors
HCC: Hepatocellular carcinoma
QB: Query block
RNA-seq: RNA (cDNA) sequencing
START: Signal track analytical research tool
SQL: Structured query language
STQL: Signal track query language
