# Supplementary material for: START: a system for flexible analysis of hundreds of genomic signal tracks in few lines of SQL-like queries
Source: BMC Genomics. 2017 Sep 22;18:749. doi: 10.1186/s12864-017-4071-1 (PMC5610441; doi:10.1186/s12864-017-4071-1)
Supplement: Supplementary file 1 — Supplementary Materials. (PDF 198 kb) [file 12864_2017_4071_MOESM1_ESM.pdf]

Supplementary Materials

Sample queries

To illustrate the use of STQL in performing practical analyses, here we describe a number of complete sample queries, including both simple ones involving single statements and composite ones involving multiple statements. Each of these queries can be tested by pasting the whole statement(s) into the query box on the main page of START and submitting the query from there.

Simple queries

SQ1 Analysis task: To compute the average H3K4me1 signal at each 100bp bin across the whole genome, for identifying potential transcriptional enhancers.

Query template:

```
SELECT   *
FROM     (project T on generate bins with length 100
with vd_sum using EACH MODEL) NtInt
WHERE    NtInt.value > 0;
```

Example of real data:

- T: ‘wgEncodeBroadHistone‘.
‘wgEncodeBroadHistoneGm12878H3k04me1StdSigV2.bigWig‘ (An ENCODE ChIP-seq data file of H3K4me1 signals in the GM12878 cell line produced by the Broad Institute)

Explanations: This is a simple demonstration of the second form of the **project on** statement. In the bigWig file we use, the intervals are all non-overlapping. In this case, using **vd_sum**, **vd_avg**, **vd_product**, **vd_max** and **vd_min** would all give the same results.

SQ2 Analysis task: To compute the expression level of each gene, defined as the average RNA (cDNA) sequencing (RNA-seq) signals covering the genomic locations of the gene.

Query template:

```
SELECT   *
FROM     (project T1 on (
         SELECT   DISTINCT chr, chrstart, chrend
         FROM     T2
         WHERE    feature = 'gene') NtInt1
         with vd_avg using EACH MODEL) NtInt2
WHERE    NtInt2.value > 0;
```

Example of real data:

- T_1: ‘wgEncodeCshlLongRnaSeq‘.
‘wgEncodeCshlLongRnaSeqGm12878CellTotalPlusRawSigRep1.bigWig‘ (An ENCODE RNA-seq data file of total long RNA in the GM12878 cell line produced by the Cold Spring Harbor Laboratory)
- T_2: ‘wgEncodeGencode‘.‘gencode.v19.annotation.gtf‘ (Gencode [21] version 19 annotation file)

Explanations: In this query, a nested query is first used to select the sequence elements in the gene annotation file that correspond to genes. “feature” is a non-default attribute defined for the gene annotation track. A projection is then performed to compute the average RNA-seq signal of each gene, and the genes with non-zero expression are returned.

SQ3 Analysis task: To find the genomic regions covered by signal peaks of both H3K4me1 and H3K27ac, which are potential active enhancers in a particular context (the HCT116 human cell line in this case).

Query template:

```
SELECT   *
FROM     T1 intersectjoin T2;
```

Example of real data:

- T_1: ‘wgEncodeSydhHistone‘.
‘wgEncodeSydhHistoneHct116H3k04me1UcdPk.narrowPeak‘ (An ENCODE ChIP-seq data file of H3K4me1 signal peaks in the HCT116 cell line produced by the Stanford/Yale/Davis/Harvard subgroup)
- T_2: ‘wgEncodeSydhHistone‘.
‘wgEncodeSydhHistoneHct116H3k27acUcdPk.narrowPeak‘ (An ENCODE ChIP-seq data file of H3K27ac signal peaks in the HCT116 cell line produced by the Stanford/Yale/Davis/Harvard subgroup)

Explanations: This query demonstrates the use of the **intersectjoin** construct in finding common regions in different signal tracks.

SQ4 Analysis task: To identify expressed regions outside annotated level-1 (experimentally validated) and level-2 (manually curated) Gencode protein-coding genes, some of which could be non-coding RNAs.

Query template:

```
SELECT  *
FROM    T1 exclusivejoin (
        SELECT  chr, chrstart, chrend
        FROM    T2
        WHERE   feature = 'gene' AND
                attributes LIKE '%gene_type "protein_coding"%'
                AND
                (attributes LIKE '%level 1%' OR
                attributes LIKE '%level 2%')
        ) NtInt;
```

Example of real data:

- T_1: 'wgEncodeCshlLongRnaSeq'. 'wgEncodeCshlLongRnaSeqGm12878CellTotalPlusRawSigRep1.bigWig' (An ENCODE RNA-seq data file of total long RNA in the GM12878 cell line produced by the Cold Spring Harbor Laboratory)
- T_2: 'wgEncodeGencode'.'gencode.v19.annotation.gtf' (Gencode version 19 annotation file)

Explanations: This query demonstrates the use of the **exclusivejoin** construct in excluding regions. A nested query is used to select out only level-1 and level-2 protein coding genes from an annotation file, based on the non-default attribute "attributes" defined for the gene annotation track. These regions are then excluded from the expressed regions with RNA-seq signals. One could also easily modify the query to exclude also small flanking regions from each gene, by selecting for example "T_2.chrstart-1000" and "T_2.chrend +1000" in the nested query, or by considering only regions with RNA-seq signals higher than a certain threshold as expressed, by pre-filtering T_1 using the WHERE clause.

SQ5 Analysis task: To identify contiguous genomic regions with significant expression, which could correspond to transcribed exons.

Query template:

```
SELECT  *
FROM    coalesce (
        SELECT  chr, chrstart, chrend, value
        FROM    T
        WHERE   value > 2) NtInt
        with vd_avg using EACH MODEL;
```

Example of real data:

- T: 'wgEncodeCshlLongRnaSeq'. 'wgEncodeCshlLongRnaSeqGm12878CellTotalPlusRawSigRep1.bigWig' (An ENCODE RNA-seq data file of total long RNA in the GM12878 cell line produced by the Cold Spring Harbor Laboratory)

Explanations: This query demonstrates the use of the **coalesce** construct in joining overlapping and adjacent regions. A nested query is used to select genomic locations with an expression level larger than 2 (say in RPKM or other units). These regions are then joined together into larger contiguous regions by using **coalesce** .

SQ6 Analysis task: To identify regions bound by a transcription factor that overlap binding sites of another factor, which could indicate co-binding events and provide information for finding functionally related factors.

Query template:

```
SELECT  *
FROM    T1 TInt1, T2 TInt2
WHERE   TInt1 overlaps with TInt2;
```

Example of real data:

- T_1: 'wgEncodeSydhTfbs'. 'wgEncodeSydhTfbsHelas3CfosStdPk.narrowPeak' (An ENCODE ChIP-seq data file of Cfos binding signal peaks in the HeLa-S3 cell line produced by the Stanford/Yale/Davis/Harvard sub-group)

- T_2: ‘wgEncodeSydhTfbs‘.
‘wgEncodeSydhTfbsHelas3CjunStdPk.narrowPeak‘ (An ENCODE ChIP-seq data file of Cjun binding signal peaks in the HeLa-S3 cell line produced by the Stanford/Yale/Davis/Harvard sub-group)

Explanations: This query demonstrates the use of the **overlaps with** relation in the WHERE clause. The query returns Cfos binding peaks that overlap Cjun binding peaks. These two factors are both members of the AP-1 complex and are expected to have overlapping binding peaks. This query is different from taking an **intersectjoin** between the two tracks (which is another possible way to study co-binding events), because **intersectjoin** only returns the overlapping parts of the intervals but not whole Cfos binding peaks.

SQ7 Analysis task: To identify all annotated genes longer than a given length.
Query template:

```
SELECT   *
FROM     T TInt
WHERE    feature = 'gene' AND length(TInt) > 1000;
```

Example of real data:

- T: ‘wgEncodeGencode‘.‘gencode.v19.annotation.gtf‘ (Gencode version 19 annotation file)

Explanations: This query demonstrates the use of the **length()** function in the WHERE clause in filtering intervals. By changing the conditions in the WHERE clause, this query could also be used for identifying other types of sequence element.

SQ8 Analysis task: To count the number of annotated non-protein-coding genes, which is relatively more variable than the number of protein-coding genes among different annotation sets and different versions of the same annotation set.
Query template:

```
SELECT   COUNT(*)
FROM     T
WHERE    feature = 'gene' AND
         attributes NOT LIKE '%gene_type "protein_coding"%';
```

Example of real data:

- T: ‘wgEncodeGencode‘.‘gencode.v19.annotation.gtf‘ (Gencode version 19 annotation file)

Explanations: This query demonstrates the use of the **COUNT()** function in the SELECT clause in computing an aggregated value of the resulting intervals. The selection condition in the WHERE clause also demonstrates how the NOT LIKE construct can be used to filter out protein coding genes from the results.

Composite queries

CQ1 Analysis task: To count the number of transcription factors with a binding peak overlapping each genomic location. Neighboring locations with the same count are grouped into one single interval in the results. This query can be used as one step in identifying regions with high occupancy of transcription-related factors (HOT) [8].
Query template:

```
FOR TRACK T IN (category=<track-category>, <track-selection-conditions>)
SELECT   chr, chrstart, chrend, value
FROM     T
COMBINED WITH UNION ALL AS Step1Results;

SELECT   *
FROM     discretize Step1Results with vd_sum using EACH MODEL;
```

Example of real data:

- <track-category>: ‘SYDH TFBS‘ (ENCODE transcription factor binding signals from ChIP-seq experiments produced by the Stanford/ Yale/ Davis/ Harvard sub-group)
- <track-selection-conditions>: cell=’GM12878’ and fname LIKE ’%Pk%’ (considering only peak files from the cell line GM12878)

Explanations: The first sub-query demonstrates the use of the FOR TRACK IN () construct in selecting all files corresponding to transcription factor binding peaks in a particular cell line. The union of all these

peaks is stored in a temporary track called Step1Results. Each of these peaks has a value of 1. In the second sub-query, the **discretize** operation is used to cut the overlapping peaks into non-overlapping regions. The number of different transcription factors with a binding peak overlapping each resulting region is counted by using the **vd_sum** operation with the **EACH MODEL** of interval values. The final results are stored in a signal track called Step2Results using the second form of CREATE TRACK.

CQ2 Analysis task: To identify regions that 1) have active transcription factor binding, 2) are not within pre-defined promoter-proximal regulatory modules and 3) are at least 10kb away from high-confidence annotated genes. These regions are potentially gene-distal regulatory regions.

Query template:

```
CREATE TRACK Step1Results AS
SELECT    NtInt_A.chr, NtInt_A.chrstart, NtInt_A.chrend
FROM      (T_1 exclusivejoin T_2) NtInt_A;

CREATE TRACK Step2Results AS
SELECT    NtInt_B.chr, NtInt_B.chrstart, NtInt_B.chrend
FROM      Step1Results NtInt_B, T_3 TInt_3
WHERE     TInt_3.feature = 'gene' AND
          (TInt_3.attributes LIKE '%level 1%' OR
          TInt_3.attributes LIKE '%level 2%') AND
          distance(NtInt_B, TInt_3) < 10000;

SELECT    *
FROM      Step1Results exclusivejoin Step2Results;
```

Example of real data:

- T_1: ‘HumanMetaTracks‘.‘BAR_Gm12878_merged.bed‘ (Regions with active transcription factor binding in GM12878 as defined in Yip et al. (2012) [8])
- T_2: ‘HumanMetaTracks‘.‘PRM_Gm12878_merged.bed‘ (Promoter-proximal regulatory regions in GM12878 as defined in Yip et al. (2012) [8])
- T_3: ‘wgEncodeGencode‘.‘gencode.v19.annotation.gtf‘ (Gencode version 19 annotation file)

Explanations: The first sub-query uses **exclusivejoin** to select regions with active transcription factor binding but are not within the pre-defined promoter-proximal regulatory regions. The second sub-query takes these regions and identifies those that are within 10,000bp from any level-1 or level-2 annotated genes in Gencode. The third sub-query removes the gene-proximal regions obtained in sub-query 2 from the regions obtained in sub-query 1 to get the final results. We designed three sub-queries for this task, rather than one single complex query (which is possible), to keep each sub-query short and easily understandable.

CQ3 Analysis task: To identify transcription factor binding regions, in the form of 100bp bins, that are at least 10kb from any high-confidence annotated genes. This is another way to identify potential gene-distal regulatory regions when the binding-active regions and the promoter-proximal regulatory modules are not pre-defined and it is desirable to give 100bp bins as outputs for further analyses.

Query template:

```
FOR TRACK T IN (category=<track-category>, <track-selection-conditions>)
SELECT    chr, chrstart, chrend, value
FROM      T
COMBINED WITH UNION ALL AS Step1Results;

CREATE TRACK Step2Results AS
SELECT    NtInt_A.chr, NtInt_A.chrstart, NtInt_A.chrend
FROM      (project Step1Results on
          generate bins with length 100
with vd_sum using EACH MODEL) NtInt_A
WHERE     NtInt_A.value > 0;

CREATE TRACK Step3Results AS
```

```
SELECT    NtInt_B.chr, NtInt_B.chrstart, NtInt_B.chrend
FROM      T_1 TInt_1, Step2Results NtInt_B
WHERE     TInt_1.feature = 'gene' AND
          (TInt_1.attributes LIKE '%level 1%' OR
          TInt_1.attributes LIKE '%level 2%') AND
          distance(NtInt_B, TInt_1) < 10000;

SELECT    *
FROM      coalesce (
          SELECT    NtInt_C.chr, NtInt_C.chrstart, NtInt_C.chrend
          FROM      (Step2Results exclusivejoin Step3Results) NtInt_C
          ) NtInt_D;
```

Example of real data:

- <track-category>: ‘SYDH TFBS‘ (ENCODE transcription factor binding signals from ChIP-seq experiments produced by the Stanford/ Yale/ Davis/ Harvard sub-group)
- <track-selection-condition>: cell=’GM12878’ and fname LIKE ’%Pk%’ (considering only peak files from the cell line GM12878)
- T_1: ‘wgEncodeGencode‘.‘gencode.v19.annotation.gtf‘ (Gencode version 19 annotation file)

Explanations: The first sub-query stores all transcription factor binding peaks in a temporary track. The second sub-query maps these regions to 100bp bins, and counts the number of transcription factors with a peak overlapping each bin. By using the “.value > 0” condition, only bins with at least one binding transcription factor are kept. The third sub-query identifies the bins that are close to level-1 or level-2 Gencode genes. Finally, the fourth sub-query uses **exclusivejoin** to find bins far away from these genes, and join those that are adjacent into larger regions.

CQ4 Analysis task: To identify genomic regions, in the form of 2000bp bins, that overlap the binding peaks of at least 2 transcription factors. The average H3K27ac signal at each of the identified regions is then computed. Thresholding the resulting signals gives a list of regions with exceptionally strong H3K27ac signals, which could be potential super enhancers.

Query template:

```
FOR TRACK T IN (category=<track-category>, <track-selection-conditions>)
SELECT    NtInt_A.chr, NtInt_A.chrstart, NtInt_A.chrend, NtInt_A.value
FROM      (project T on
          generate bins with length 2000
with vd_sum using EACH MODEL) NtInt_A
WHERE     NtInt_A.value > 0
COMBINED WITH UNION ALL AS Step1Results;

CREATE TRACK Step2Results AS
SELECT    chr, chrstart, chrend, COUNT(*) AS value
FROM      Step1Results
GROUP BY chr, chrstart, chrend;

CREATE TRACK Step3Results AS
SELECT    chr, chrstart, chrend
FROM      Step2Results
WHERE     value > 2;

CREATE TRACK Step4Results AS
SELECT    NtInt_B.chr, NtInt_B.chrstart, NtInt_B.chrend, NtInt_B.value
FROM      (project T on Step3Results
with vd_sum using EACH MODEL) NtInt_B;

SELECT    *
FROM      Step4Results
WHERE     value > 3;
```

Example of real data:

- <track-category>: ‘SYDH TFBS‘ (ENCODE transcription factor binding signals from ChIP-seq experiments produced by the Stanford /Yale /Davis /Harvard sub-group)
- <track-selection-conditions>: cell=’K562’ and fname LIKE ’%Pk%’ (considering only peak files from the cell line K562)
- T: ‘wgEncodeBroadHistone‘. ‘wgEncodeBroadHistoneK562H3k27acStdSig.bigWig‘ (An ENCODE ChIP-seq data file of H3K27ac signals in the K562 cell line produced by the Broad Institute)

Explanations: In the first sub-query, all peak files of transcription factor binding from a particular cell line are selected. Each of them is projected onto 2000bp bins, so that a bin has value 1 if it overlaps with a binding peak, or value 0 if it does not. Only bins that overlap with at least one binding peak are kept. In the second sub-query, the number of transcription factors with a binding peak overlapping a bin is counted by using the **COUNT()** function and the GROUP BY clause. In the third sub-query, only bins that overlap with at least the binding peaks of a certain number of (e.g., 2) different transcription factors are kept. In the fourth sub-query, H3K27ac signals are mapped onto these remaining bins. Finally, in the fifth sub-query, only bins with an H3K27ac level larger than a threshold (e.g., 3) are kept in the output. Again, it is possible to write the STQL statements in a more compact form, but separating them into sub-queries makes each one easy to write and to understand.

CQ5 Analysis task: To identify genes with significant differential binding signals at their promoters in two different contexts. In each context, the binding signals are computed by subtracting the ChIP-seq signals by the corresponding background signals obtained from a control experiment.

Query template:

CREATE TRACK Step1Results AS
SELECT chr, chrstart, chrend, strand
FROM T_1
WHERE feature = ’gene’ AND
attributes LIKE ’%gene_type “protein_coding”%’;

CREATE TRACK Step2Results AS
SELECT DISTINCT $NtInt_A$.chr, $NtInt_A$.chrstart, $NtInt_A$.chrend
FROM (SELECT chr, chrstart-1500 AS chrstart,
chrstart +500 AS chrend
FROM Step1Results
WHERE strand = ’+’
UNION ALL
SELECT chr, chrend-500 AS chrstart,
chrend +1500 AS chrend
FROM Step1Results
WHERE strand = ’-’) $NtInt_A$;

CREATE TRACK Step3Results AS
SELECT $NtInt_B$.chr, $NtInt_B$.chrstart, $NtInt_B$.chrend,
$NtInt_B$.value - $NtInt_C$.value as value
FROM (**project** T_2 **on** Step2Results
with vd_sum using EACH MODEL) $NtInt_B$,
(**project** T_3 **on** Step2Results
with vd_sum using EACH MODEL) $NtInt_C$
WHERE $NtInt_B$ **coincides with** $NtInt_C$;

CREATE TRACK Step4Results AS
SELECT $NtInt_D$.chr, $NtInt_D$.chrstart, $NtInt_D$.chrend,
$NtInt_D$.value - $NtInt_E$.value as value
FROM (**project** T_4 **on** Step2Results
with vd_sum using EACH MODEL) $NtInt_D$,

```
                  (project T_5 on Step2Results
with vd_sum using EACH MODEL) NtInt_E
WHERE     NtInt_D coincides with NtInt_E;

CREATE TRACK Step5Results AS
SELECT    NtInt_F.chr, NtInt_F.chrstart, NtInt_F.chrend,
          NtInt_F.value/ NtInt_G.value as value
FROM      Step3Results NtInt_F,
          (SELECT   chr, chrstart, chrend, value
          FROM      Step4Results
          WHERE     value != 0) NtInt_G
WHERE     NtInt_F coincides with NtInt_G;

CREATE TRACK Step6Results AS
SELECT    chr, chrstart, chrend
FROM      Step5Results
WHERE     value > 2;

SELECT    *
FROM      (SELECT   NtInt_H.chr, NtInt_H.chrstart, NtInt_H.chrend,
                    NtInt_H.strand
          FROM      Step1Results NtInt_H,
                    (SELECT   chr, chrstart +1500 AS chrstart,
                              chrstart +1500 AS chrend
                    FROM      Step6Results) NtInt_I
          WHERE     NtInt_H.strand = '+' AND
                    NtInt_I is prefix of NtInt_H
          UNION ALL
          (SELECT   NtInt_J.chr, NtInt_J.chrstart, NtInt_J.chrend,
                    NtInt_J.strand
          FROM      Step1Results NtInt_J,
                    (SELECT   chr, chrend-1500 AS chrstart,
                    chrend-1500 AS chrend
                    FROM      Step6Results) NtInt_K
          WHERE     NtInt_J.strand = '-' AND
                    NtInt_K is suffix of NtInt_J) NtInt_L;
```

Example of real data:

- T_1: ‘wgEncodeGencode‘.‘gencode.v19.annotation.gtf‘ (Gencode version 19 annotation file)
- T_2: ‘wgEncodeSydhTfbs‘.
  ‘wgEncodeSydhTfbsGm12878JundIggrabSig.bigWig‘ (An ENCODE ChIP-seq data file of Cjun binding signals in the GM12878 cell line produced by the Stanford/Yale/Davis/Harvard sub-group)
- T_3: ‘wgEncodeSydhTfbs‘.
  ‘wgEncodeSydhTfbsGm12878InputStdSig.bigWig‘ (An ENCODE control experiment file using input DNA in the GM12878 cell line produced by the Stanford/Yale/Davis/Harvard sub-group)
- T_4: ‘wgEncodeSydhTfbs‘.
  ‘wgEncodeSydhTfbsK562JundIggrabSig.bigWig‘ (An ENCODE ChIP-seq data file of Cjun binding signals in the K562 cell line produced by the Stanford/Yale/Davis/Harvard sub-group)
- T_5: ‘wgEncodeSydhTfbs‘.
  ‘wgEncodeSydhTfbsK562InputStdSig.bigWig‘ (An ENCODE control experiment file using input DNA in the K562 cell line produced by the Stanford/Yale/Davis/Harvard sub-group)

Explanations: The first sub-query identifies all protein-coding genes. The second sub-query defines the promoter of each gene as the region from 1500bp upstream of the transcription start site to 500bp downstream of it. The two strands need to be handled in different ways. The third and fourth sub-queries compute the background-subtracted binding signals of a transcription factor at the promoters in two different cell lines.

The fifth sub-query computes the fold change of the binding signal, given that the signal is non-zero in the second cell line. The sixth sub-query selects the promoters with at least a 2-fold higher binding signal in the first cell line as compared to the second one. Finally, the seventh sub-query gets back the information of the genes of these promoters.

Since the results of the first two sub-queries are frequently used, they can be pre-constructed for reuse by various queries, which would simplify the whole analysis procedure. START allows users to store their custom tracks, which will be explained in the next section.

CQ6 Analysis task: To identify genomic regions with bi-directional transcription at their flanking regions (Figure S1), which could be potential enhancers producing enhancer RNAs (eRNAs) [29, 30].

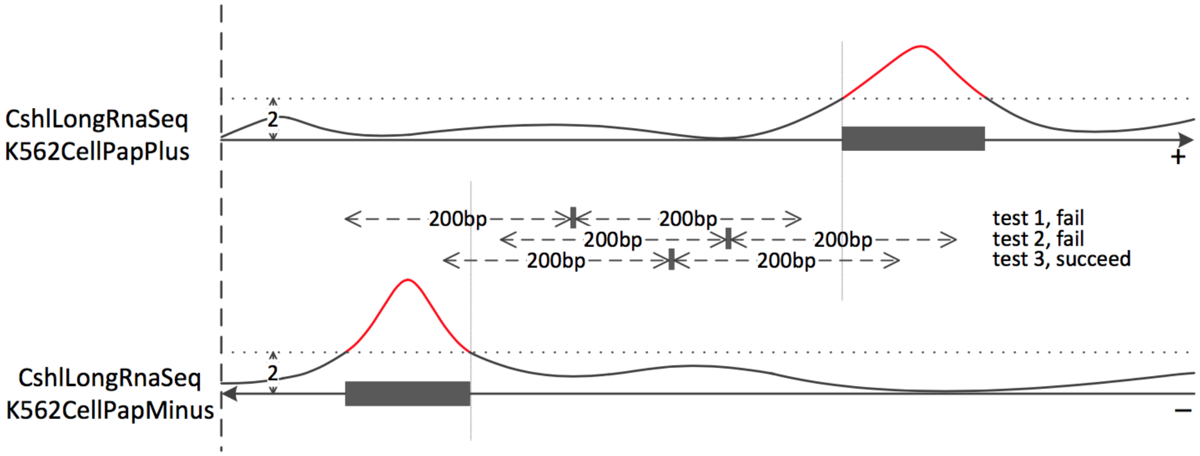

Figure S1 Illustration of the regions to be identified in this query. RNA-seq signals on the two strands are shown in the two tracks. In the middle we show three genomic regions in the form of vertical bars. The first and second regions do not satisfy the query requirements since they miss significant RNA-seq signals on either side, while the third region satisfies them by having significant signals on both sides. This query identifies all regions that satisfy the requirements in the whole genome.

Query template:

```
CREATE TRACK Step1Results AS
SELECT    chr, chrstart - 200 AS chrstart, chrend - 200 AS chrend
FROM      T1
WHERE     value > 2;

CREATE TRACK Step2Results AS
SELECT    chr, chrstart + 200 AS chrstart, chrend + 200 AS chrend
FROM      T2
WHERE     value > 2;

SELECT    *
FROM      Step1Results intersectjoin Step2Results;
```

Example of real data:

- T_1: ‘wgEncodeCshlLongRnaSeq‘.
  ‘wgEncodeCshlLongRnaSeqK562CellPapPlusRawSigRep1.bigWig‘ (An ENCODE RNA-seq data file of total long RNA of the positive strand in the K562 cell line produced by the Cold Spring Harbor Laboratory)
- T_2: ‘wgEncodeCshlLongRnaSeq‘.
  ‘wgEncodeCshlLongRnaSeqK562CellPapMinusRawSigRep1.bigWig‘ (An ENCODE RNA-seq data file of total long RNA of the negative strand in the K562 cell line produced by the Cold Spring Harbor Laboratory)

Explanations: In the first sub-query, genomic regions on the positive strand with an expression level higher than a given value (e.g., 2) are selected. The regions are shifted 200bp to the left, which will make the

last step easy. Likewise, the second sub-query identifies regions on the negative strand with significant expression, and the regions are shifted to the right by 200bp. Finally, in the third sub-query, the results from the first two sub-queries are intersected. For each region in the final signal track, every constituent genomic position has significant expression level 200bp downstream on the positive strand and 200bp upstream on the negative strand, which forms a bi-directional pattern indicative of eRNA [29].

Comparisons between STQL and SQL

Since STQL has an SQL-like syntax and a data model that is essentially a relation, one may wonder whether STQL queries can be easily expressed in SQL. In this section, we use four examples to show that some operations are much more difficult to perform using SQL than STQL.

Ex.1 The first example involves the **is closest to each** construct. In STQL, it is easy to find out the interval(s) in track T_2 closest to each interval in track T_1 using the following query:

```
SELECT    T1.chr, T1.chrstart AS start1, T1.chrend AS end1, T2.chrstart AS start2, T2.chrend AS end2
FROM      T1 TInt1, T2 TInt2
WHERE     TInt1 is closest to each TInt2;
```

To perform the same operation in SQL, three steps are needed, namely 1) computing the distance of all interval pairs from the two tracks, 2) finding the minimum distance for each interval in T_1, and 3) retrieving the corresponding pairs with these minimum distances:

```
WITH
/* 1. Calculate the distance between all pairs of intervals from T1 and T2 */
T1sDistance AS (
SELECT    T1.chr, T1.chrstart AS start1, T1.chrend AS end1, T2.chrstart AS start2, T2.chrend AS end2,
          CASE /* Different cases for calculating distance between two intervals */
          WHEN T1.chrstart <= T2.chrend AND T1.chrend >= T2.chrstart
                    THEN 0
          WHEN T1.chrend < T2.chrstart
                    THEN T2.chrstart - T1.chrend
          WHEN T1.chrstart > T2.chrend
                    THEN T1.chrstart - T2.chrend
          END AS distance
FROM      T1, T2
WHERE     T1.chr = T2.chr
),

/* 2. Calculate the minimum interval distance of each interval in T1 */
T1sMinDistance AS (
          SELECT    chr, start1, end1, min(distance) AS minDistance
          FROM      T1sDistance
          GROUP BY chr, start1, end1
)

/* 3. Find out the closest pairs based on the minimum distances */
SELECT    *
FROM      T1sDistance a
          LEFT JOIN T1sMinDistance b
                    ON a.chr = b.chr
                    AND a.start1 = b.start1
                    AND a.end1 = b.end1
WHERE     distance = minDistance
```

Ex.2 The second example involves the **coalesce** construct. Sample query SQ5 demonstrates how all the genomic positions with certain level of transcription signals are merged into disjoint regions using **coalesce** . Since each interval needs to be combined with an indefinite number of other intervals to form an output region, the operation cannot be performed using standard SQL. We wrote the following SQL query involving recursion to handle this task:

```
WITH RECURSIVE /* Recursively coalesce the intervals */
CoalesceGroup AS (
SELECT      chr, chrstart, chrend
FROM T
WHERE T.value > 2
UNION
SELECT      a.chr, a.chrstart, T.chrend
FROM        CoalesceGroup AS a
/* Join overlapping intervals */
JOIN c on    a.chr = c.chr
            AND a.chrstart <= c.chrend + 1 AND a.chrend >= c.chrstart - 1
WHERE       c.value > 2
),
CoalesceGroup2 AS (
SELECT      *, row_number() OVER (PARTITION BY chr, chrend ORDER BY chrstart) AS rn
FROM        CoalesceGroup
)

/* Compute the values of the output intervals */
SELECT      a.chr, a.chrstart, a.chrend, AVG(c.value) as value
FROM        (
            SELECT      chr, chrstart, MAX(chrend) AS chrend
            FROM        CoalesceGroup2
            WHERE       rn = 1
            GROUP BY chr, chrstart) a
            LEFT JOIN c ON a.chrstart <= c.chrend AND a.chrend >= c.chrstart
WHERE       c.value > 2
GROUP BY a.chr, a.chrstart, a.chrend
```

Ex.3 The third example involves the **discretize** construct. STQL can be used to discretize the intervals in a track into non-overlapping intervals:

```
SELECT      *
FROM        discretize T with vd_sum using EACH MODEL
```

The same operation can be performed by the following SQL query:

```
/* Determine the non-overlapping intervals */
WITH allPos AS (
SELECT      row_number() OVER (PARTITION BY chr ORDER BY pos) AS rn, chr, pos
FROM        ((
                        SELECT chr, d.chrstart AS pos FROM d GROUP BY chr, pos
                        UNION
                        SELECT chr, d.chrend+1 AS pos FROM d
                        WHERE d.chrend != (SELECT MAX(d.chrend) FROM d) GROUP BY chr, pos
            )
            UNION ALL
            (
                        SELECT chr, d.chrstart-1 AS pos FROM d
                        WHERE d.chrstart != (SELECT MIN(d.chrstart) FROM d) GROUP BY chr, pos
                        UNION
                        SELECT chr, d.chrend AS pos FROM d GROUP BY chr, pos
            )) tmpUnion
),
```

```
grouping AS (
SELECT      a.chr, a.pos AS chrstart, b.pos AS chrend
FROM        allPos a LEFT JOIN allPos b ON a.rn+1 = b.rn AND a.chr = b.chr
WHERE       a.rn % 2 = 1)

/* Compute the values of the output intervals */
SELECT      a.chr, a.chrstart, a.chrend, SUM(d.value) AS value
FROM        grouping a JOIN d ON a.chr = d.chr AND a.chrstart <= d.chrend AND a.chrend >= d.chrstart
GROUP BY a.chr, a.chrstart, a.chrend
```

Ex.4 The last example invovles the **project on generate bins with length** constructs. Sample query SQ1 demonstrates how the average signal within each 100bp genomic bin can be easily computed using these constructs. To perform the same operation in SQL, it has to first define a new table consisting of the bin definitions, and then compute the average signal value in each bin:

```
/* 1. Create the bin definitions using the GENERATE_SERIES function in PostgreSQL */
WITH
Bin AS (
SELECT'chr1' AS chr, c.b+1 AS chrstart, c.b+100 AS chrend FROM GENERATE_SERIES(0, 249250621, 100) c(b)
UNION ALL
SELECT'chr2' AS chr, c.b+1 AS chrstart, c.b+100 AS chrend FROM GENERATE_SERIES(0, 243199373, 100) c(b)
UNION ALL
SELECT'chr3' AS chr, c.b+1 AS chrstart, c.b + 100 AS chrend FROM GENERATE_SERIES(0, 198022430, 100) c(b)
UNION ALL
SELECT'chr4' AS chr, c.b+1 AS chrstart, c.b + 100 AS chrend FROM GENERATE_SERIES(0, 191154276, 100) c(b)
UNION ALL
SELECT'chr5' AS chr, c.b+1 AS chrstart, c.b + 100 AS chrend FROM GENERATE_SERIES(0, 180915260, 100) c(b)
UNION ALL
SELECT'chr6' AS chr, c.b+1 AS chrstart, c.b + 100 AS chrend FROM GENERATE_SERIES(0, 171115067, 100) c(b)
UNION ALL
SELECT'chr7' AS chr, c.b+1 AS chrstart, c.b + 100 AS chrend FROM GENERATE_SERIES(0, 159138663, 100) c(b)
UNION ALL
SELECT'chr8' AS chr, c.b+1 AS chrstart, c.b + 100 AS chrend FROM GENERATE_SERIES(0, 146364022, 100) c(b)
UNION ALL
SELECT'chr9' AS chr, c.b+1 AS chrstart, c.b + 100 AS chrend FROM GENERATE_SERIES(0, 141213431, 100) c(b)
UNION ALL
SELECT'chr10' AS chr, c.b+1 AS chrstart, c.b + 100 AS chrend FROM GENERATE_SERIES(0, 135534747, 100) c(b)
UNION ALL
SELECT'chr11' AS chr, c.b+1 AS chrstart, c.b + 100 AS chrend FROM GENERATE_SERIES(0, 135006516, 100) c(b)
UNION ALL
SELECT'chr12' AS chr, c.b+1 AS chrstart, c.b + 100 AS chrend FROM GENERATE_SERIES(0, 133851895, 100) c(b)
UNION ALL
SELECT'chr13' AS chr, c.b+1 AS chrstart, c.b + 100 AS chrend FROM GENERATE_SERIES(0, 115169878, 100) c(b)
UNION ALL
SELECT'chr14' AS chr, c.b+1 AS chrstart, c.b + 100 AS chrend FROM GENERATE_SERIES(0, 107349540, 100) c(b)
UNION ALL
SELECT'chr15' AS chr, c.b+1 AS chrstart, c.b + 100 AS chrend FROM GENERATE_SERIES(0, 102531392, 100) c(b)
UNION ALL
```

```
SELECT'chr16' AS chr, c.b+1 AS chrstart, c.b + 100 AS chrend FROM GENERATE_SERIES(0, 90354753, 100) c(b)
UNION ALL
SELECT'chr17' AS chr, c.b+1 AS chrstart, c.b + 100 AS chrend FROM GENERATE_SERIES(0, 81195210, 100) c(b)
UNION ALL
SELECT'chr18' AS chr, c.b+1 AS chrstart, c.b + 100 AS chrend FROM GENERATE_SERIES(0, 78077248, 100) c(b)
UNION ALL
SELECT'chr19' AS chr, c.b+1 AS chrstart, c.b + 100 AS chrend FROM GENERATE_SERIES(0, 59128983, 100) c(b)
UNION ALL
SELECT'chr20' AS chr, c.b+1 AS chrstart, c.b + 100 AS chrend FROM GENERATE_SERIES(0, 63025520, 100) c(b)
UNION ALL
SELECT'chr21' AS chr, c.b+1 AS chrstart, c.b + 100 AS chrend FROM GENERATE_SERIES(0, 48129895, 100) c(b)
UNION ALL
SELECT'chr22' AS chr, c.b+1 AS chrstart, c.b + 100 AS chrend FROM GENERATE_SERIES(0, 51304566, 100) c(b)
UNION ALL
SELECT'chrX' AS chr, c.b+1 AS chrstart, c.b + 100 AS chrend FROM GENERATE_SERIES(0, 155270560, 100) c(b)
UNION ALL
SELECT'chrY' AS chr, c.b+1 AS chrstart, c.b + 100 AS chrend FROM GENERATE_SERIES(0, 59373566, 100) c(b)
)

/* 2. Compute average signal value of each bin */
SELECT    *
FROM      (
          SELECT    Bin.chr, Bin.chrstart, Bin.chrend,
                    SUM(CASE /* Different cases based on the intersection between an interval an a bin */
                    WHEN Bin.chrstart > T.chrstart AND Bin.chrend > T.chrend
                              THEN T.chrend - Bin.chrstart + 1
                    WHEN Bin.chrstart <= T.chrstart AND Bin.chrend > T.chrend
                              THEN T.chrend - T.chrstart + 1
                    WHEN Bin.chrstart > T.chrstart AND Bin.chrend <= T.chrend
                              THEN Bin.chrend - Bin.chrstart + 1
                    WHEN Bin.chrstart <= T.chrstart AND Bin.chrend <= T.chrend
                              THEN Bin.chrend - T.chrstart + 1
                    END * T.value)/(Bin.chrend - Bin.chrstart + 1) AS value
          FROM      Bin LEFT JOIN T on /* Join all overlapping intervals and bins */
                    (Bin.chrstart <= T.chrend and Bin.chrend >= T.chrstart AND Bin.chr = T.chr)
          GROUP BY Bin.chr, Bin.chrstart, Bin.chrend ) AS Ntint
WHERE     Ntint.value > 0
```
